# Supplementary material for: Discovery and application of insertion-deletion (INDEL) polymorphisms for QTL mapping of early life-history traits in Atlantic salmon
Source: BMC Genomics. 2010 Mar 8;11:156. doi: 10.1186/1471-2164-11-156 (PMC2838853; doi:10.1186/1471-2164-11-156)
Supplement: Additional file 2 — Information on developed 76 locus single-run INDEL panel in Atlantic salmon. Information on fluorescence labeling, primer concentrations, PCR pooling and links to alignments, INDEL motifs and GENESCAN (Burge and Karlin 1997) predictions of genes/exons are available in html format. [file 1471-2164-11-156-S2.ZIP › Additionalfile2/snpsummary21965.html]

```
Cluster 12384 Contig 1

prev  Summary    Contig List  next
```

Size of Consensus sequence = 1116

Number of sequences = 4

Minimum redundancy = 1

Key

A gi|89865619|gb|DY721742.1|DY721742 EST\_ssal\_rgb2\_77481 ssalrgb2 mixed\_tissue Salmo salar cDNA Salmo salar cDNA clone ssal\_rgb2\_627\_070\_rev 5', mRNA sequence  
B gi|117829573|gb|EG902269.1|EG902269 EST\_ssal\_evf\_40944 ssalevf mixed\_tissue Salmo salar cDNA Salmo salar cDNA clone ssal\_evf\_554\_253\_rev 5', mRNA sequence  
C gi|117829574|gb|EG902270.1|EG902270 EST\_ssal\_evf\_40945 ssalevf mixed\_tissue Salmo salar cDNA Salmo salar cDNA clone ssal\_evf\_554\_253\_fwd 3', mRNA sequence  
D gi|89836713|gb|DY696270.1|DY696270 EST\_ssal\_rgb2\_52009 ssalrgb2 mixed\_tissue Salmo salar cDNA Salmo salar cDNA clone ssal\_rgb2\_584\_067\_rev 5', mRNA sequence

7 SNPs detected

A B C D  cosegregation weighted

675 . C C -   7/7 75.00
676 . A A -   7/7 75.00
677 . G G -   7/7 75.00
678 . T T -   7/7 75.00
679 . T T -   7/7 75.00
680 . G G -   7/7 75.00
995 . . G C   7/7 50.00
